# Stacking Interventions Enhances Carbon Removals and Profitability of Livestock Production Systems

**DOI:** 10.1002/advs.202503382

**Published:** 2025-06-25

**Authors:** My Pham‐Kieu, Stephen Ives, Warwick Badgery, Matthew Tom Harrison

**Affiliations:** ^1^ Tasmania Institute of Agriculture University of Tasmania Launceston TAS 7248 Australia; ^2^ New South Wales Department of Primary Industries Orange NSW 2800 Australia; ^3^ Vietnam National University of Agriculture Hanoi 12406 Vietnam

**Keywords:** co‐benefit, manure, nitrous oxide, optimization, organic carbon, sequestration

## Abstract

While previous studies have primarily examined the impacts of singular interventions on GHG emissions reduction and carbon dioxide removals (CDR), few studies explore complementarities and antagonisms when multiple interventions are simultaneously operationalized. Here, the aim is to examine how stacking of two pathways for mitigation–CDR, via soil organic carbon (SOC) accrual and GHG emissions avoidance via antimethanogenic feed additives–impacts net GHG emissions associated with sheep production. A nonlinear programing approach is invoked to elicit optimal combinations of grazing management and antimethanogenic feed additives to maximize farm profit and/or minimize net GHG emissions. It is shown that stacking multiple interventions realizes that greater abatement and profit do any singular intervention. Adoption of 3‐NOP feed supplement with 15‐ and 30‐paddock high stocking rate systems is the most prospective stacked intervention for concurrent profit maximization and emissions minimization. It is contended that (1) increasing payments for farming of carbon and ecosystems services relative to that of wool and meat will stimulate participation in carbon markets, (2) economics of participation in carbon markets tend to be more favorable for larger farms than smaller farms due to economies of scale and (3) adoption of optimal grazing management and antimethanogenic feed additives can realize more profit from sheep production and carbon farming than enterprises that only derive income from sheep production.

## Graphical abstract

The study indicates that stacking multiple interventions aimed at maximising soil organic carbon (SOC) sequestration and enteric methane (CH4) reductions realizes greater abatement and profit do any singular intervention, especially when SOC sequestration accounts for a significant proportion of greenhouse gas emissions (GHG) mitigation.

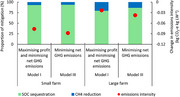

## 1 Introduction

Sheep production has long been a key pillar of the Australian agricultural sector,^[^ ^1^, ^2^ ^]^ with wool and sheep meat production respectively worth $2.9B and $4.6B ($ refers to Australian Dollars) in 2024–25.^[^ ^3^ ^]^ Such production has however been a source of greenhouse gas (GHG) emissions, particularly due to methane from ruminant enteric fermentation.^[^ ^4^, ^5^, ^6^ ^]^ There is thus a need to conceive, operationalize, and refine mitigation strategies that appropriately balance economic viability, environmental stewardship, social responsibility, and food/fiber security.^[^ ^7^, ^8^ ^]^ While opportunities for emissions mitigation are subject to enterprise mix, production system, and agroecological region,^[^ ^9^, ^10^, ^11^ ^]^ mitigation interventions can be conceptually compartmentalized into those that either remove carbon dioxide (CO_2_) from the atmosphere–known as carbon dioxide removals (CDR)–or those that reduce or avoid GHG emissions, known as emissions reduction. While emissions reduction and CDR are often examined using a reductionist lens, viz.^[^ ^12^, ^13^ ^]^ few studies examine interdisciplinary outcomes associated with stacking or bundling mitigation innovations.^[^ ^5^, ^14^, ^15^, ^16^ ^]^ E.g., a study in south‐eastern Australia^[^ ^17^ ^]^ showed that while few singular interventions to cattle production systems enhanced productivity and profitability while also reducing GHG emissions; adoption of seaweed (*Asparagopsis* spp.) as a livestock feed supplement and planting trees realized the greatest mitigation relative to status quo operations (67%–95%) due to combined carbon sequestration in trees and GHG emissions reduction. The study^[^ ^17^ ^]^ also showed that enterprise revenue diversification with wind turbines and adoption of livestock genotypes with improved feed‐conversion efficiency were the most conducive to economic gains (17%–39%). These observations suggest that economic, environmental and agrifood co‐benefits and trade‐offs associated with purported innovations should be carefully considered prior to implementation.^[^ ^16^ ^]^

Soil organic carbon (SOC) sequestration is a key avenue for CDR^[^ ^18^, ^19^, ^20^, ^21^, ^22^ ^]^ as incremental gains in SOC at scale have substantial potential for CDR.^[^ ^23^, ^24^ ^]^ Improved grazing management, such as implementing higher stocking rates for shorter period in concert with longer rest durations between grazing events have been mooted for improving SOC sequestration,^[^ ^25^, ^26^ ^]^ although evidence for such grazing regimes improving SOC over the long‐term is lacking. Improved soil organic matter can also emanate productivity co‐benefits such as improved soil fertility and improved soil health, which improve profit and natural resources on farm.^[^ ^27^, ^28^ ^]^ While alternative grazing management practices are effective in theory, their success depends on many factors, such as the vicissitudes of the weather,^[^ ^26^ ^]^ historical management,^[^ ^16^ ^]^ and current SOC stocks.^[^ ^24^ ^]^ While many studies have considered mitigation potential; externalities, such as cost, have significant bearing on whether or not adoption will occur, spread and/or perpetuate.^[^ ^13^, ^29^ ^]^ As such, costs associated with paddock division into smaller areas–as is common for cell grazing systems–are likely to be expensive due to need for fencing, infrastructure, and labor,^[^ ^30^, ^31^ ^]^ particularly on large farms.

As enteric methane generally comprises the largest source of direct farm GHG in the absence of land‐use change, many practices and technologies have been proposed for enteric methane inhibition. E.g., much research has been conducted on the implications associated with antimethanogenic feed additives viz.,^[^ ^32^, ^33^, ^34^, ^35^ ^]^ such as *Asparagopsis taxiformis*,^[^ ^36^, ^37^, ^38^, ^39^, ^40^, ^41^, ^42^ ^]^ 3‐NOP,^[^ ^43^, ^44^, ^45^, ^46^, ^47^ ^]^ antimethanogenic pastures^[^ ^6^, ^48^ ^]^ and biochar^[^ ^49^, ^50^, ^51^, ^52^, ^53^ ^]^ on livestock production and/or enteric methane. *Asparagopsis*,^[^ ^54^, ^55^ ^]^ 3‐NOP,^[^ ^44^, ^56^, ^57^ ^]^ and biochar^[^ ^58^, ^59^ ^]^ have been shown to reduce enteric methane by 34–99%, 21–86%, 0.5–47%, respectively. *Asparagopsis* and 3‐NOP are considered highly efficacious in enteric methane abatement^[^ ^60^, ^61^, ^62^ ^]^ although it must be acknowledged that more data is available from scientifically controlled environments where dietary content is strictly regulated compared with measurements of enteric methane from livestock in free‐range grazing systems. In contrast to production systems where feed can be manipulated on a daily basis, such as dairies, piggeries and feedlots, pragmatic connotations associated with daily feed supplementation of livestock in extensive grazing scenarios remain an open question. Cattle in the extensive rangelands of northern Australia may only be mustered twice per year, e.g., suggesting that provision of a daily feed supplement in such production systems would be unrealistic.^[^ ^63^ ^]^ Logistical constraints associated with regular sourcing of feed additives may further limit feasibility, as could cumulative costs associated with need to deliver feed supplements on a regular basis, and reduce efficiency due to less frequent supplement. These observations suggest that costs of feed supplementation need to be weighed up against other avenues for CDR or GHG emissions reduction using a whole of systems lens. As such, our objectives were to:

1. Contrast SOC accrual and economic viability conferred by various grazing management approaches;
2. Contrast the quantum of mitigation and profit associated with various feed additives for reducing enteric methane, and
3. Determine optimal management regimes with an objective of maximizing profit and/or minimizing GHG emissions.

This study advances the state of the art in many ways; through (1) contrasting the relative change in net farm GHG emissions with SOC accrual versus mitigation of enteric methane, (2) by contrasting management approaches that maximize economic performance versus those that aspire for optimal environmental outcomes, (3) examining how stacking of multiple reduction and removal interventions impacts economic and environmental outcomes and (4) quantifying how optimal management approaches change dynamically.

## 2 Experimental Section

**Figure 1:**
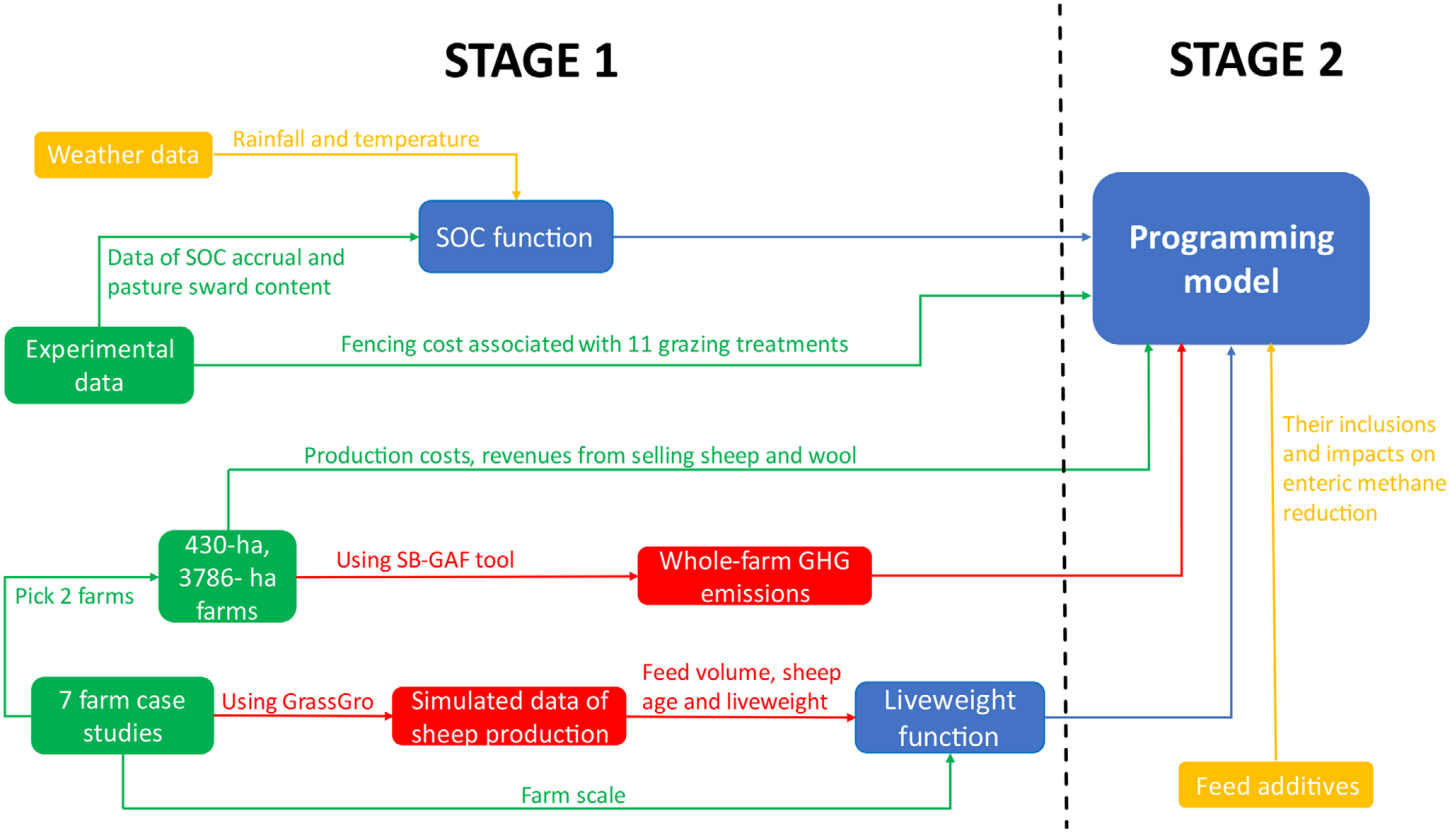
Conceptual design of the study. Green represents input data from peer‐reviewed data experiment and farm case studies. Red represents simulated data used as an input to the programing model, yellow represents published data from SILO^[^ ^64^ ^]^ or peer‐reviewed papers,^[^ ^43^, ^56^, ^58^, ^59^, ^65^ ^]^ while blue represents the programing model structure and constraints. Fencing costs were based on those of multi‐paddock systems.^[^ ^30^ ^]^ Stage 1 represents initialization computations, while Stage 2 represents the optimization of grazing management and feed additive decisions to maximize profit and/or minimize GHG emissions.

This study includes two key stages as shown in **Figure** **1**. The first stage relates to inputs for programing models, including creating functions for SOC accrual, sheep liveweight gain, and whole farm GHG emissions. The second stage requires using outputs from these functions in programing models to compare impacts of all plausible farm decisions and optimized management for minimizing GHG emissions, maximizing profit, or both. For the first stage, empirical data from ten‐year field experiments were used to simulate relationships between SOC accrual with sequestration duration, pasture sward content, grazing management regimes and weather, with the objective of determining how those relationships affected adoption decisions. Following the workflow articulated previously,^[^ ^70^ ^]^ these experiments were extrapolated to the farm scale (Section 2.2), a programing approach (Section 2.3) was then evoked to elicit optimal management scenarios assuming that farms could adopt one or more grazing treatments across the farm area. Details of SOC data and weather conditions are given in Section 2.1. Seven farm case studies were used in the first stage to analyze impacts of sheep production on adoption decisions. Outputs from the model GrassGro were used to construct a production frontier of sheep liveweight for each farm (see Section 2.2 for details). From this group of seven, one small‐scale and one large‐scale farms were selected for further comparison. For this, whole farm GHG emissions and emissions intensity were calculated using the sheep‐beef greenhouse accounting framework (SB‐GAF; Section 2.2). All inputs, including raw and simulated data, were then added to the programing model (Stage 2). Formulation of the programing models and their scenarios are detailed in Section 2.3. These models aimed to determine the trade‐off between farm profit and GHG abatement by optimizing management across 11 grazing treatments (Section 2.1) and 7 combinations of antimethanogenic feed additives (Section 2.3.3).

### 2.1 Site Characteristics

Data pertaining to pastures, SOC stocks and botanical composition were sourced from long‐term field experiments conducted in New South Wales, Australia, from 2012 to 2021.^[^ ^67^ ^]^ The site was located on Red Dermosol soil (phosphorus P = 57 mg kg^−1^, and pH_CaCl2_ = 6.4) with three‐year‐old pasture comprising cocksfoot (*Dactylis glomerata* L., varieties Porto and Kara) as the dominant grass, and white clover (*Trifolium repens* L.) as the dominant legume. Weather data for ten years of the experiment were sourced from SILO.^[^ ^64^ ^]^ Over the experimental duration, average annual rainfall was 913 mm year^−1^, while average annual minimum and maximum temperatures were 7 °C and 19 °C, respectively. Following peer‐reviewed literature,^[^ ^66^, ^67^ ^]^ 11 grazing treatments that were conducted with 12‐month‐old Merino wethers, using alternative numbers of paddocks, stocking rates and rest durations (**Table** **1**), were examined. Experimental and weather data were used to construct a SOC function, which represents the relationship between SOC stocks and sequestration period, pasture sward content, weather and grazing treatments, which was then included as a key constraint of the programing models (constraint 2, Section 2.3). The dataset included observations across ten years, with each treatment conducted in a randomized complete block design.^[^ ^66^, ^67^ ^]^

**Table 1:** Grazing management and paddock structure derived from field experiments conducted in New South Wales, Australia^[^ ^66^, ^67^ ^]^ from 2012 to 2021. One dry sheep equiv. (DSE) represents a 50 kg two‐year‐old castrated male Merino animal consuming 7.6 MJ day^⁻1^.

Grazing management	Details
Number of paddocks	‐ One‐paddock system ‐ 15‐paddock system ‐ 30‐paddock system
Annual stocking rates	‐ High stocking rate: 13.6 DSE ha^⁻1^ ‐ Low stocking rate: 8.8 DSE ha^⁻1^ ‐ Flexible grazing: Average 9.2 DSE ha^⁻1^ over the experimental duration, sheep were moved on and off pasture based on feed availability.
Paddock rest duration	‐ Continuous grazing: paddocks were not given any rest from grazing. ‐ Fast rotation: average rest period between subsequent grazing events was 56 days, each event went for 2–4 days, and grazing was conducted six times annually. ‐ Slow rotation: average rest duration was 112 days, 4–8 days of continuous grazing, with each paddock grazed three times per annum.

### 2.2 Sheep Production and GHG Emissions Data

Experimental and simulated data of sheep production systems were invoked as inputs of the programing models (Figure 1). Experimental data (farm size, stock categories, revenues, production costs) were sourced from seven real sheep farm case studies in Australia. These farms were chosen from a national selection process, based on their location within dominant national sheep production zones, farm size, production system (e.g., self‐replacing), likelihood of continued participation and ability to implement interventions. To simulate sheep production, the model GrassGro (https://grazplan.csiro.au/grassgro/) was used to predict biophysical interactions between pasture growth, animal performance, and weather conditions.^[^ ^71^, ^72^ ^]^ This process began by inputting long‐term daily climate data (sourced from SILO, https://www.longpaddock.qld.gov.au/silo/), including rainfall, temperature, solar radiation, and evaporation for each farm location. Soil parameters in each location were defined using the soil Atlas within GrassGro and refined with farmer knowledge. All feeds, including fresh pasture (dominant pasture species, seasonal growth rates, pasture composition), supplementary feeds (types, amount, feeding schedule), and farm management (replacement animal, animal sales) were used as GrassGro inputs. GrassGro simulations were ran for all farms over a 40‐year period on a daily basis (from 1 July 1982 to 30 June 2022), generating key outputs such as sheep liveweight, sheep age, pasture availability, animal intake, and supplementary feed requirements. These outputs were then used to construct the liveweight function in the programing model (constraint 3, Section 2.3) for each of the seven farms.

**Figure 2:**
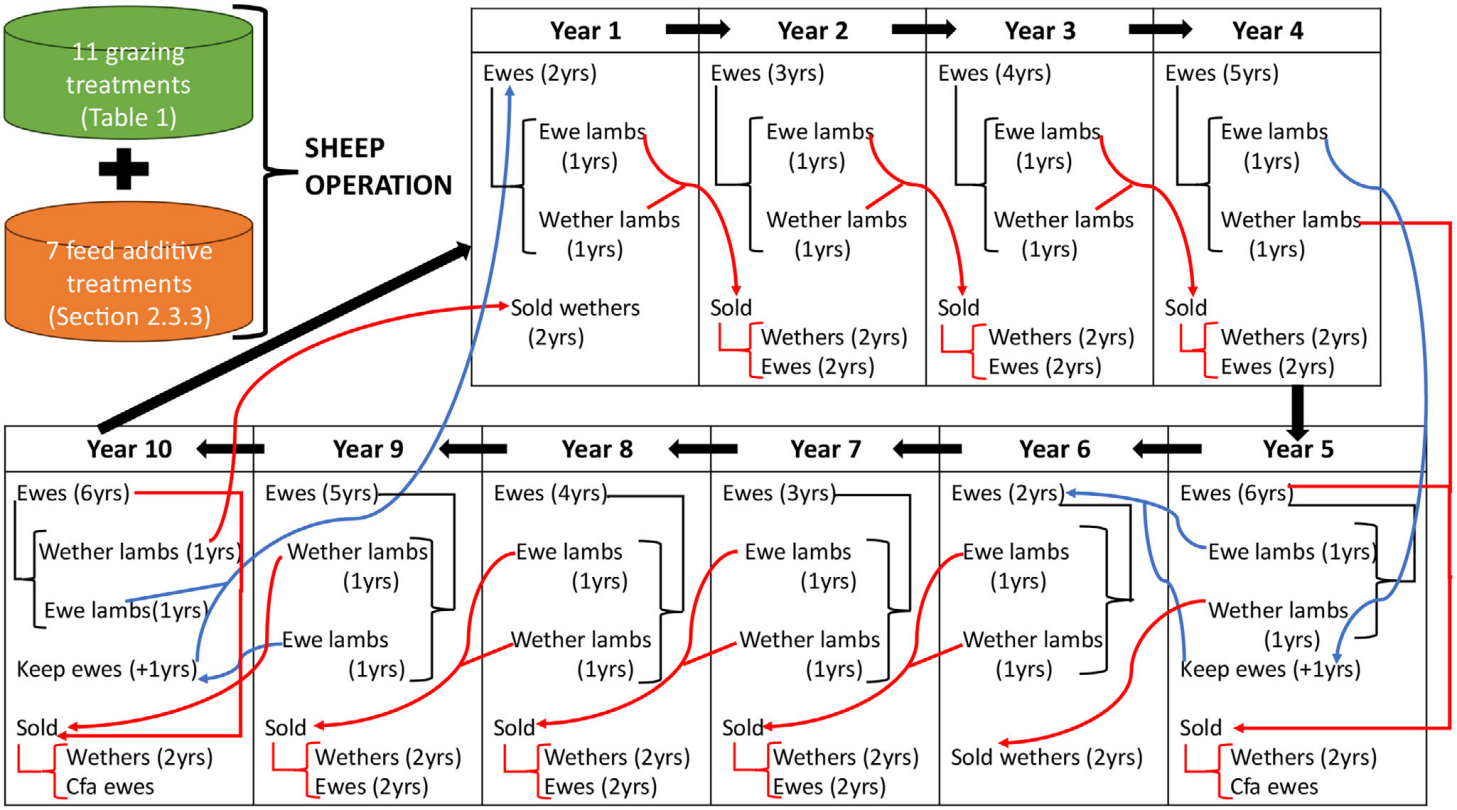
Self‐replacing flock dynamics used in each programing model. Simulations were conducted with Merino ewes joined to terminal sires. Sheep sold include ewes and wethers at two years, while cast for age (CFA) ewes represent ewes culled from the main breeding flock after five years. Blue arrows represent ewe replacement cohorts, while red arrows represent cohorts destined for sale. Black arrows represent main breeding flock activities, such as lambing of breeding ewes, two‐year‐old ewes in year one becoming three‐year‐old ewes in year two et seq.

Of seven farms, two located in New South Wales, Australia–one 430 hectares and the other 3786 hectares–were selected for deeper analysis due to regional proximity. Given that farm size influences GHG, carbon removals and income, a rational question is to examine how farm scale impacts on optimal management depending on whether GHG mitigation, profit, or mitigation and profit was the main objective. The two farms chosen were not intended to represent the full diversity of Australian sheep farms, but to provide insight as to how variation in farm size influences optimal management. By selecting two real farms within the same agroecological region, the study obviated the effect of climate or soil on results. Farm inputs, such as electricity, fuel, fertilizer, and GrassGro outputs for the two farms, such as sheep numbers, liveweight, diet quality, feed supplementary requirements, meat and wool production, were entered into the sheep‐beef greenhouse accounting framework (SB‐GAF v2.2, https://piccc.org.au/resources/Tools) to quantify total GHG emissions (CO_2_‐equivalent ha⁻^1^), which is based on Australian National Greenhouse Inventory guidelines. SB‐GAF accounts for total GHG emissions across Scope 1, 2, and 3 categories for each farm baseline. Scope 1 emissions included direct farm sources, such as manure and enteric methane, nitrous oxide emissions from urine, dung, and fertilizer, and carbon dioxide from electricity and fuel use. Scope 2 emissions represented indirect emissions from the generation of electricity purchased and used on farms. Scope 3 emissions captured other indirect emissions associated with upstream production and transportation of supplementary feeds and fertilizers. Total GHG emissions were allocated based on the mass of protein in meat produced, then divided by sheep liveweight to calculate emission intensity (kg CO_2_‐e kg LW⁻^1^). Characteristics pertaining to the two sheep farms, including area, stock categories (**Figure** **2**), revenue from sheep meat and wool, and production costs (such as land management, sales, stock management, pasture management, supplementary feeding, and fixed costs) were added as constraints of the programing models. Further details of production costs are given in the supporting information. It is assumed that a core flock of breeding ewes was maintained on farm for six years, with breeding ewes between two and six years of age lambing annually, being cast for age at six years (Figure 2). For simplicity and to standardize assumptions across treatments, zero mortality, a lamb weaning rate of 100%, and equal birth rates of ewes and wether lambs, were assumed.

### 2.3 Programing Model

A nonlinear programing paradigm was adopted to identify grazing management and antimethanogenic feed additive combinations that maximize profit and/or minimize GHG by iterating through various farm areas subject to a given treatment (Table 1), number of sheep sold, and sheep age. The model was developed for a self‐replacing flock with breeding ewes replaced by ewe lambs born within the enterprise every sixth year (Figure 2). Where the goal was maximum profit and/or minimum GHG, it was assumed that the farm would adopt simultaneous grazing and feed management interventions for ten years, and would adopt one or more grazing‐ and/or antimethanogenic feed treatments in each year. A ten‐year production cycle was used to align with the experimental duration of SOC sequestration data to elicit deeper insight into long‐term trade‐offs between profitability and GHG emissions. Sheep production and land use were interlinked in the model. The number of sheep influenced land use via the duration and stocking rate of each treatment, while availability and productivity of land in turn constrained maximum flock size through pasture growth and carrying capacity. These interactions were dynamically adjusted depending on whether the optimization objective was profit, emissions reduction, or both. This approach ensured that land use and grazing management were concurrently optimized each year, reflecting real‐world interplay between stocking decisions and land condition.

#### 2.3.1 Model Initialization

Frontier functions of SOC sequestration and sheep productivity (liveweight) were formulated for each empirical model (Section 2.3.2). The frontier regression method was used to quantify dependant variables, being SOC accrual (*Y_SOC_*) and sheep liveweight (*Y_sheep_*). The SOC function was constructed using experimental data (Section 2.1). Following derivation of the model for examining trade‐offs between farm profit and SOC sequestration associated with grazing management,^[^ ^70^ ^]^ independent variables were compartmentalized in four groups: weather, biology, sequestration duration, and management. Relationships between SOC accrual and sequestration duration, weather and botanical composition associated with 11 grazing treatments were next included in programing models.

- The “Weather” category accounted for variability in annual rainfall (*CV_rainfall_*) and temperature (*CV_temperature_*) of the experimental location^[^ ^66^ ^]^ from 2012 to 2021. *CV_rainfall_* [%] and *CV_temperature_* [%] were estimated by dividing annual standard deviation of rainfall and temperature by their mean values respectively. Rainfall and temperature data were derived from SILO,^[^ ^64^ ^]^ while coefficients for *CV_rainfall_* and *CV_temperature_* were determined following peer‐reviewed literature:^[^ ^73^ ^]^
$$\mathrm{Weather}=\left(0.67\times CV_{\mathrm{rainfall}}\right)+\left(0.33\times CV_{\mathrm{temperature}}\right)$$
- The “biology” group, including grass and legume pasture species, were assumed continuous variables, following SOC experimental data.^[^ ^66^ ^]^
- “Carbon sequestration duration” was matched with the flock replacement cycle (Figure 2).
- “Management” included dummy variables (*G1, … G10*), representing combinations of paddock numbers, stocking rates and rest duration used to capture effects of 11 grazing treatments on SOC flux. This approach accounted for variable SOC sequestration rates across paddocks (see **Table** **2**). In constructing the programing model, standard econometric practice was followed and one treatment (flexible grazing) was excluded as the reference category, allowing the function to capture relative effects of the remaining treatments compared with the omitted category, thereby avoiding scenarios of perfect correlation. These treatment effects were then used as inputs to the programing models to assess trade‐offs between profit and GHG emissions.

**Table 2:** Variable and parameter definitions.

Abbreviation	Variable and parameter	Dimensions
*A*	farm area used for grazing (ha), *A* = 430 ha or 3786 ha	hectares (ha)
*a_it_*	grazing area of treatment *i* in *t* years	ha
*c_jt_*	total production costs associated with feed option *j* in *t* years	^a)^$ year⁻^1^
*CC*	grass dry matter (DM)	tonnes DM ha⁻^1^
*ce_it_*	carbon price in *t* years	$ tonne CO_2_‐e⁻^1^
*e*	SOC conversion into CO_2_‐e; *e* = 3.67 CO_2_‐e tonnes SOC^−1^	CO_2_‐e tonnes SOC⁻^1^
*e_GHG_*	GHG emission intensity; based in SB‐GAF calculations for each of 430 ha and 3786 ha farms	kg CO_2_‐e kg liveweight⁻^1^ (kg CO_2_‐e kg LW⁻^1^)
*fe_it_*	fencing cost associated with treatment *i* in *t* years	$ year⁻^1^
*FP*	simulated pasture DM for sheep maintenance or growth	kg month⁻^1^ DSE⁻^1^
*FS*	simulated supplement DM for sheep growth	kg month⁻^1^ DSE⁻^1^
*G1, … G10*	dummy variables for combinations of paddock number, stocking rate and rest duration	
	*G1* = 1 represents continuous grazing‐high stocking rate, while *G1* = 0 represents otherwise	
	*G2* = 1 represents continuous grazing‐low stocking rate, while *G2* = 0 represents otherwise	
	*G3* = 1 represents 15 paddock‐fast rotation‐high stocking rate, *G3* = 0 represents otherwise	
	*G4* = 1 represents 30 paddock‐fast rotation‐high stocking rate, *G4* = 0 represents otherwise	
	*G5* = 1 represents 15 paddock‐fast rotation‐low stocking rate, *G5* = 0 represents otherwise	
	*G6* = 1 represents 30 paddock‐fast rotation‐low stocking rate, *G6* = 0 represents otherwise	
	*G7* = 1 represents 15 paddock‐slow rotation‐high stocking rate, *G7* = 0 represents otherwise	
	*G8* = 1 represents 30 paddock‐slow rotation‐high stocking rate, *G8* = 0 represents otherwise	
	*G9* = 1 represents 15 paddock‐slow rotation‐low stocking rate, *G9* = 0 represents otherwise	
	*G10* = 1 represents 30 paddock‐slow rotation‐low stocking rate, *G10* = 0 represents otherwise	
	Flexible grazing was modeled by setting all indicator variables to zero: (*G1* = *G2* = *G3* = *G4* = *G5* = *G6* = *G7* = *G8* = *G9* = *G10* = 0)	
*i*	grazing treatment; *i* = continuous grazing‐high stocking rate (coded 1), continuous grazing‐low stocking rate (coded 2), flexible grazing (coded 3), 15 paddock‐fast rotation‐high stocking rate (coded 4), 30 paddock‐fast rotation‐high stocking rate (coded 5), 15 paddock‐fast rotation‐low stocking rate (coded 6), 30 paddock‐fast rotation‐low stocking rate (coded 7), 15 paddock‐slow rotation‐high stocking rate (coded 8), 30 paddock‐slow rotation‐high stocking rate (coded 9), 15 paddock‐slow rotation‐low stocking rate (coded 10), 30 paddock‐slow rotation‐low stocking rate (coded 11); thus *i* = 1, …11	
*j*	feed option; *j* = nil feed additives (coded 1), feed with 0.25% *Asparagopsis* (coded 2), feed with 0.5% *Asparagopsis* (coded 3), feed with 0.1% 3‐NOP and 21% enteric methane reduction (coded 4), feed with 0.1% 3‐NOP and 25% enteric methane reduction (coded 5), feed with 1% walnut shell biochar (coded 6), feed with 1.5% chicken manure biochar (coded 7); hence, *j* = 1, …7	
*LE*	legume DM	tonnes DM ha⁻^1^
*m_jt_*	profit (of sheep sales) associated with feed option *j* in *t* years; *m_jt_* ≥ 0	$ year⁻^1^
*M*	objective function of total profit across the farm area	$
*n_t‐born_*	number of lambs born in *t* years	DSE year⁻^1^
*n_t‐ewe_*	number of ewes in *t* years	DSE year⁻^1^
*n_t‐sold_*	number of sheep sold in *t* years	DSE year⁻^1^
*PC*	minimum pasture cover when grazing sheep	kg DM ha⁻^1^
*r_jt_*	sheep sale revenue associated with feed option *j* in *t* years, *r_jt_* ≥ 0	$ year⁻^1^
*r_wean_*	lamb weaning rate, *r_wean_* = 1	
*re_jt_*	GHG emission reduction associated with feed option *j* in *t* years	kg CO_2_‐e kg LW⁻^1^
*rw_jt_*	wool revenue associated with feed option *j* in *t* years; *rw_jt_* ≥ 0	$ year⁻^1^
*S*	Dummy variable for farm scale; *S* = 1 represents small, *S* = 0 represents large	
*SA*	age of ewes or wethers	months old DSE⁻^1^
*t*	years of sheep production, *t* = 1, … 10	years
*T*	time of carbon sequestration used in SOC function, *T* = *t*	years
*w_jt_*	enteric methane reduction associated with feed option *j* in *t* years	tonnes CO_2_‐e
*WE*	“weather”	%
*ws_it_*	SOC accrual associated with treatment *i* in *t* years; *ws_it_* ≥ 0	tonnes
*Y_sheep_*	frontier function of sheep liveweight	kg DSE⁻^1^
*Y_SOC_*	frontier function of SOC accrual in each year	tonnes ha⁻^1^ year⁻^1^
*Z*	objective function of whole farm net GHG emissions	tonnes CO_2_‐e
*z_t_*	total GHG emissions in *t* years	tonnes CO_2_‐e

^a)^ $ refers to Australian Dollars.

Sheep liveweight, modeled as a stochastic frontier production function of sheep age, feed requirement, and farm scale, was also added in the programing models. This function was based on GrassGro outputs for the seven farms.

- Sheep age, a variable of the liveweight function (model constraint (3), Section 2.3.2), was formulated as a polynomial to account for the relationship between sheep liveweight and age.
- Feed requirement included pasture and supplementary feed for sheep maintenance or growth, varying with sheep age and liveweight. Data were sourced from GrassGro simulations for each farm (constraint 11). Pasture used for sheep growth was governed by available DM (constraint 11, Section 2.3.2).
- Farm scale (*S*) was modeled using a dummy variable. *S* = 1 or 0 for the small or large farm respectively.

#### 2.3.2 Optimization Approaches

Three empirical models were conceptualized for two representative farms to elicit optimal combinations of grazing treatments and antimethanogenic feed additives resulting in the lowest net GHG emissions (sum of CH_4_, N_2_O, CO_2_ with SOC sequestered subtracted after conversation to CO_2_‐e) and maximum farm profit. The first model (Model I) examined trade‐offs between farm profit and emissions abatement, while Models II and III were used for comparison with the first model. Those models had similar constraints but differed in their objective function. Since farms are businesses and must maintain cash flow to subsist, the constraint was adopted in which sheep revenue (*r_jt_*) and profit (*m_jt_*) must be positive (zero or higher).

- **Model I**: The aim of this model was to determine the optimal combination of grazing management and antimethanogenic feed additives to concurrently maximize profit and minimize net GHG emissions. Carbon removals with SOC accrual and mitigation of enteric methane were converted to environmental income using carbon price^[^ ^74^, ^75^ ^]^ and quantum of carbon sequestration in tonnes of CO_2_‐e^[^ ^76^ ^]^:
(1) $$\begin{array}{cc} MAX\;M & =\sum_{j=1}^{7}\sum_{t=1}^{10}m_{jt}+\sum_{j=1}^{7}\sum_{t=1}^{10}rw_{jt}+\sum_{i=1}^{11}\sum_{t=1}^{10}ws_{it}\\ & \;\times ce_{it}\times e-\sum_{j=1}^{7}\sum_{t=1}^{10}\left(z_{t}-w_{jt}\right)\times ce_{jt}-\sum_{i=1}^{11}\sum_{t=1}^{10}fe_{it} \end{array}$$

Subject to:

(2) $$\begin{array}{cc} Y_{SOC} & =f(T,LE,CC,WE,G1,G2,G3,G4,G5,\\ & \;\times G6,G7,G8,G9,G10) \end{array}$$

(3) $$Y_{\mathrm{sheep}}=f\left(SA,FP,FS,S\right)$$

(4) $$\sum_{i\;=1}^{11}\sum_{j\;=1}^{7}\sum_{t\;=1}^{10}n_{t-born}=n_{t-ewe}$$

(5) $$\sum_{i\;=1}^{11}\sum_{j\;=1}^{7}\sum_{t\;=1}^{10}n_{t-ewe}\times r_{wean}=n_{t-sold}$$

(6) $$\sum_{t\;=1}^{10}e_{GHG}\times \;Y_{sheep}\times \left(n_{t-ewe}+n_{t-sold}\right)=Z_{t}$$

(7) $$\sum_{j\;=1}^{7}\sum_{t\;=1}^{10}re_{jt}\times \;Y_{sheep}\times \left(n_{t-ewe}+n_{t-sold}\right)=w_{jt}$$

(8) $$\sum_{i\;=1}^{11}\sum_{t\;=1}^{10}a_{it}\times \;Y_{SOC}=ws_{it}$$

(9) $$\sum_{i\;=1}^{11}\sum_{t\;=1}^{10}a_{it}=A$$

(10) $$\sum_{j\;=1}^{7}\sum_{t\;=1}^{10}r_{jt}-c_{jt}=m_{jt}$$

(11) FP+PC=CC+LE

Ten constraints were applied to Equation (1): constraint (2) determines SOC accrual based on sequestration period, pasture biomass, weather and integrated effects; constraint (3) is a frontier function of sheep liveweight based on data from seven commercial farms and modeled as a function of age, feed biomass, farm scale, and interactions thereto; constraint (4) represents the number of ewes for a given grazing treatment; constraint (5) represents the number of sold sheep based on the number of ewes and lamb weaning rate; constraint (6) represents total farm GHG emissions; constraint (7) represents enteric methane reduction associated with selected feed options; constraint (8) represents whole farm SOC flux under any given grazing treatment; constraint (9) represents all grazing area subject to *i* treatments; constraint (10) represents sheep profit with revenue from selling sheep less production costs, and constraint (11) represents the relationship between green biomass, pasture cover, and pasture requirements for sheep maintenance or growth. The number of ewes and sheep sold for constraints (4) and (5) was determined based on stocking rates, farm scale and the number of breeding ewes. Revenue and production costs (management costs, feed costs, fixed costs) were calculated per DSE based on adoption of grazing treatments and antimethanogenic feed additives.

- **Model II**: Single objective function with similar constraints to the first model to identify the combination of grazing treatments and antimethanogenic feed additives that maximized profit from sheep and wool sales.
(12) $$Max\;M=\sum_{j\;=1}^{7}\sum_{t\;=1}^{10}m_{jt}+\sum_{j\;=1}^{7}\sum_{t\;=1}^{10}rw_{jt}-\sum_{i\;=1}^{11}\sum_{t\;=1}^{10}fe_{it}$$
- **Model III** was similar to Model II, but with an objective to determine optimal grazing treatment and antimethanogenic feed additive combinations that minimized net farm GHG emissions.
(13) $$Min\;Z=\sum_{j\;=1}^{7}\sum_{t\;=1}^{10}\left(z_{t}-w_{jt}\right)-\sum_{i\;=1}^{11}\sum_{t\;=1}^{10}ws_{it}$$

#### 2.3.3 Scenario Analysis

Following,^[^ ^70^ ^]^ the initial scenario was defined as the optimal combination of grazing and antimethanogenic feed additive management of each empirical model when all constraints were satisfied for all inputs, including farm area, meat and wool revenue, production costs, weather, and carbon price (set at $38 per tonne CO_2_‐e^[^ ^75^, ^77^ ^]^). It is important to distinguish between the types of supplementary feed used in this analysis. “Supplementary feed” (e.g., hay, lupins, wheat and grain) is used when pasture feeds were low; supplementary feed was invoked only to support liveweight maintenance to an average flock condition score of 3.0. Supplementary feeding automatically occurred across the simulation using GrassGro when pasture supplies were less than flock feed demand, which is common in summer/early autumn for much of southern Australia. Antimethanogenic feed additives, i.e., feed additives designed only to reduce enteric methane (biochar, *Asparagopsis* and 3‐NOP) rather than support liveweight maintenance (which was the purpose of the hay, lupins, wheat and grain), are also invoked. Costs associated with feed use were accounted for per unit mass, so systems that consumed more feed were attributed greater feed costs than those that consumed less. Constant labor costs associated with supplementary feed were used, because the labor support would be employed on farm regardless of whether or not they were feeding animals. It is assumed that purchased feed supplements such as hay, lupins, wheat, and grain (as distinct from antimethanogenic feed additives) were fed to all animals on a daily basis for maintenance of mature animals and for growth of juvenile animals. Seven antimethanogenic feed additive combinations (**Table** **3**) were formulated based on their relevance to sheep production systems, market readiness, and efficacy in enteric methane emissions inhibition. These antimethanogenic feed additives were fed with supplementary feeds on a daily basis. As use of feed supplements in extensive grazing system may be lower than that under controlled conditions, conservative enteric methane reduction was assumed for all antimethanogenic feed additives based on empirical results in peer‐reviewed studies.^[^ ^43^, ^56^, ^58^, ^59^, ^65^ ^]^ Options 2 and 3 were based on literature^[^ ^55^, ^65^, ^78^ ^]^ using *Asparagopsis taxiformis*; options 4 and 5 experiments using 3‐NOP,^[^ ^44^ ^]^ option 6 a review of biochar^[^ ^58^ ^]^ indicating that 0.5% dietary intake of biochar reduced enteric methane by 0.5%, while option 7, was based on a meta‐analysis of dietary rumen modulating strategies^[^ ^59^ ^]^ which found a 5.5% reduction with 2% biochar dietary inclusion. No effects of biochar on energetic needs (maintenance or growth) are assumed.

**Table 3:** Antimethanogenic feed additives used in the present study.

Option		Inclusion of anti‐methanogenic feed additives		Enteric methane reduction	Cost
Supplementary feed without antimethanogenic feed additives	Option 1	Not applicable	Not applicable		Cost of purchased supplementary feeds based on two selected farms (Supporting Information)
Supplementary feed with *Asparagopsis taxiformis*	Option 2	0.25% organic matter basis	No change in methane production		Cost of *Asparagopsis* was $0.16‐0.25/DSE/day.^[^ ^68^ ^]^
	Option 3	0.5% organic matter basis	34% reduction		
Supplementary feed with 3‐NOP	Option 4	0.1% organic matter basis	21% reduction		Cost of 3‐NOP was $0.09 DSE⁻^1^ day⁻^1^.^[^ ^69^ ^]^
	Option 5	0.1% organic matter basis	25% reduction		
Supplementary feed with biochar	Option 6	0.5% organic matter basis	0.5% reduction		Cost of biochar was $2 kg⁻^1^.^[^ ^49^ ^]^
	Option 7	2% organic matter basis	5.5% reduction		

To examine the sensitivity of the conclusions to economic assumptions, four sheep prices, four supplement costs, four pasture costs, and three carbon price scenarios were examined. Baseline sheep prices were sourced from two commercial farms (see Supporting Information), and by way of sensitivity analysis were increased or decreased by 20% or 50% based on historical ranges observed in long term sheep prices.^[^ ^79^ ^]^ Four supplementary feed cost and pasture labor cost scenarios were also examined, in each case by either increasing or decreasing costs by 25% or 75%. Two future carbon prices ($50 and $100 per t CO_2_‐e^[^ ^49^, ^75^ ^]^) were also compared with the present carbon spot price ($38 per t CO_2_‐e).

#### 2.3.4 Statistical Analysis

Stochastic frontier functions were invoked to examine relationships between SOC accrual with sequestration duration, sward pasture content, weather and grazing management (constraint 2); or associations between sheep liveweight with age, feed intake and farm scale (constraint 3). Distributions of continuous variables underpinning the SOC function were assessed to confirm that they were normally distributed using the Shapiro‐Wilk test. Parameters in the SOC accrual and sheep liveweight functions were computed using maximum likelihood estimation. A Wald chi‐square test was used to test overall significance and Z‐scores of explanatory variables; all statistical tests were two‐sided with significant levels of 0.01, 0.05, or 0.1 (see results). Sample sizes varied according to the text and treatment and are presented in the results (Section 3). All analyses were carried out using Stata version 18.0.

## 3 Results

### 3.1 Association between SOC Accrual, Sequestration Period, Pasture Biomass, Grazing Management and Weather

Greater SOC accrual occurred with longer sequestration duration, although short term declines in SOC occurred due to drought. Although sward grass and legume content had little influence on SOC, their interaction with sequestration period and/or weather had greater impacts. Our purpose in determining the relationship between SOC stocks and sequestration duration, weather and pasture biomass was so that we could then elucidate optimal grazing treatments (Section 3.3). Sward grass and legume content as key factors in the programing model were associated with pasture biomass (constraint 11, Section 2.3.2), which influenced sheep liveweight **Table** **4**.

**Table 4:** Regression coefficients associated with SOC sequestration sourced from field experiments (*n* = 99) in New South Wales, Australia.^[^ ^66^, ^67^ ^]^

Variable	Coefficient	Z‐score
Intercept^a)^	4.41	1.48
Sequestration period (years)	0.72	2.86***
Legume content (tonnes DM ha⁻^1^)	22.13	0.64
Cocksfoot content (t DM ha⁻^1^)	0.82	0.51
Weather (%)	− 2.99	− 3.26***
*G1*	− 1.90	− 1.32
*G2*	− 1.61	− 1.27
*G3*	0.71	0.58
*G4*	− 1.41	− 1.21
*G5*	− 2.02	− 1.76*
*G6*	− 0.68	− 0.57
*G7*	− 0.56	− 0.47
*G8*	− 0.18	− 0.15
*G9*	0.26	0.22
*G10*	− 2.59	− 2.06**
Weather × Legume	118.41	4.05***
Sequestration period × Legume	− 23.21	− 3.31***
Wald test	44.00	
Log likelihood function	− 228.07***	

^a)^ SOC accrual (tonnes ha^−1^ year^−1^) was the dependent variable

***, **, * represent significance at the 1%, 5%, and 10% levels respectively; coefficients without an asterisk are not significant.

### 3.2 Association between Liveweight, Sheep Age, Feed Intake, and Farm Scale

Sheep age had a significant effect on liveweight. Feed intake was positively related to liveweight, pasture biomass, and supplementary feed intake. Farm scale also had significant impacts on liveweight per DSE (**Table** **5**). Relationships between sheep liveweight and sheep age, supplementary feed and farm scale were next used to determine optimal antimethanogenic feed additives and calculate emissions intensity on small and large farms (Section 3.3).

**Table 5:** Coefficients and Z‐scores of variables associated with sheep productivity (*n* = 2637). All variables are significant at the 1% level. DM = dry matter.

Variable	Coefficient	Z‐score
Intercept^a)^	2.9605	723
Sheep age	0.0520	284
Sheep age^2^	− 0.0005	− 140
Ln(Pasture DM)	0.0058	13
Ln(Supplement DM)	0.0019	7
Farm scale	0.0089	14
Wald test	910 188	
Log likelihood function	7503***	

^a)^ Ln(liveweight) was the dependent variable

### 3.3 Optimal Grazing and Feed Management

**Figure 3:**
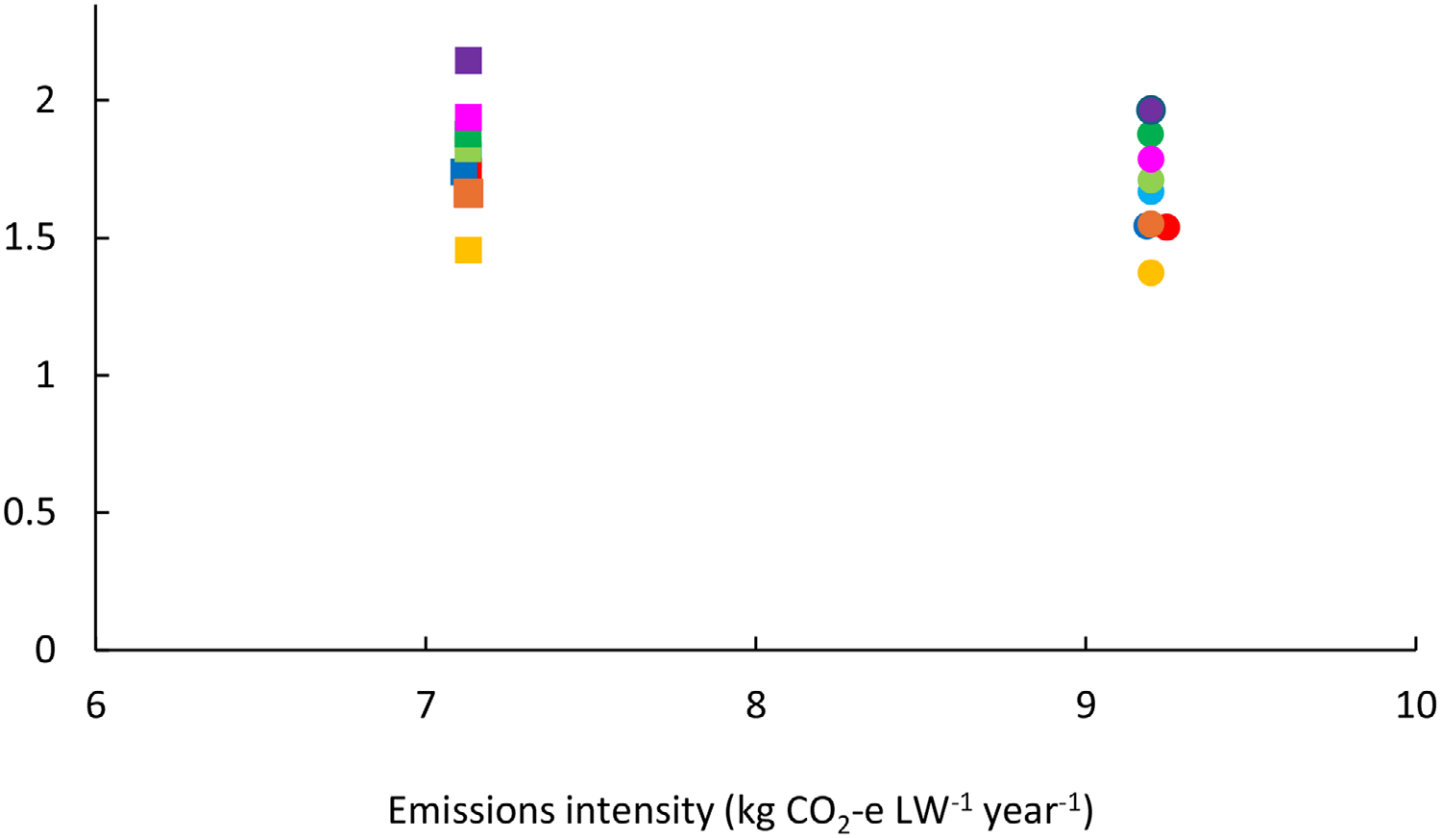
Trade‐offs between profit and GHG emissions reduction. Circles represent the small farm, squares represent the large farm. Light blue, red and dark blue depict results from Models I, II, and III, respectively, for the initial scenario. Model I optimized the combination of grazing treatment and antimethanogenic feed additives to maximize profit and minimize net GHG emissions, simultaneously. Model II optimized grazing treatment and antimethanogenic feed additives to maximize profit from sheep and wool sales. Model III optimized grazing treatment and low‐emission feed additives to minimize net GHG emissions. Models II and III included profit from sheep and wool sales, while total profit of Model I included income from sheep and wool production as well as GHG emission reduction, assuming a carbon price of $38 per t CO2‐e. Light and dark green points depict carbon price scenarios at $50 and $100 per t CO_2_‐e, respectively. Yellow, orange, pink, and purple points depict four sheep price scenarios (decreasing 20%, 50% or increasing 20%, 50% respectively). Dark blue, orange and red circles overlap on the small farm, while light green, dark blue, red and light blue squares overlap on the large farm.

**Figure 4:**
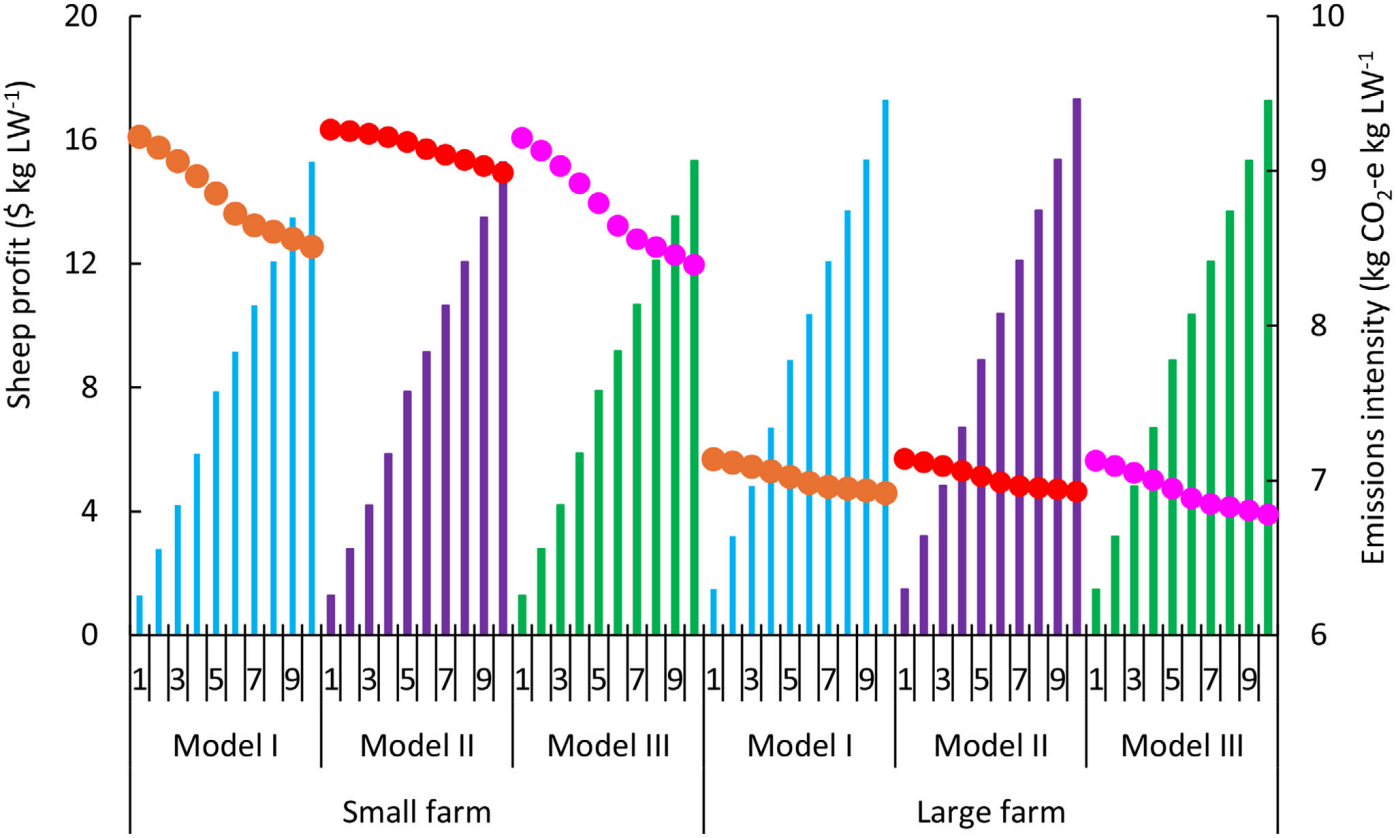
Cumulative profit and emissions intensity for three optimized management scenarios for small and large farms. Numbers on x‐axis represent years. Model I optimized grazing management and feed additives to maximize total profit and minimize net GHG emissions, simultaneously. Model II optimized grazing management and feed additives to maximize profit from sheep and wool sales. Model III optimized grazing management and feed additives to minimize net GHG emissions. Blue, purple, and green bars represent cumulative profit from Models I, II, and III, respectively. Orange, red, and pink circles represent cumulative emissions intensity from Models I, II, and III respectively.

Multiple grazing treatments (except for continuous grazing) stacked with antimethanogenic feed additives were shown to comprise optimal solutions for both farms. De minimus grazing area was allocated to either the 15‐paddock system with fast rotation and low stocking rate or the 30‐paddock system with slow rotation and low stocking rate, despite strong relationships with SOC accrual. Optimal solutions for the small farm were closer to the objective of Model III compared with those for the large farm (**Figures** **3**,**4**). Emissions intensity trends were similar between Models I and III for the small farm whereas they were similar among Models I and II for the large farm (Figure 4); likely due to differences in management options, production costs and price between two farms. Despite greater emissions reduction achieved, absolute emissions intensity for the small farm was higher than those for the large farm due to higher initial level (Figure 3). For the small farm, Model I resulted in $0.4 DSE⁻^1^ lower profit and 2.3 t CO_2_‐e DSE⁻^1^ lower emissions intensity than that from Model II. Over the simulation, trends in annual profit and emissions intensity primarily reflected flock management, with culled animals sold in years when emissions intensity decreased.

**Figure 5:**
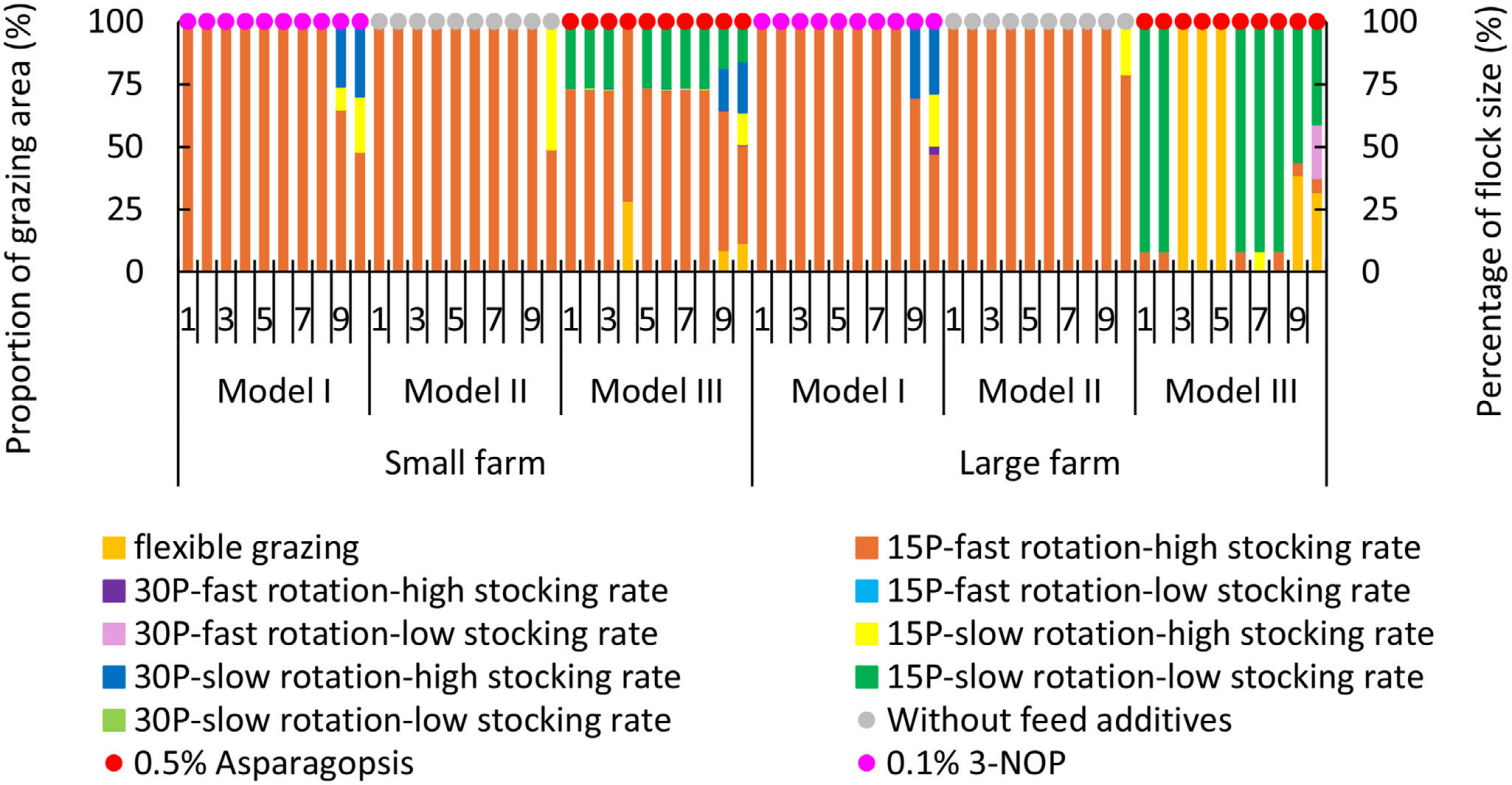
Optimal grazing and antimethanogenic feed management regimes. Carbon price of Model I was set at $38 per t CO_2_‐e; 15P and 30P represent 15‐ and 30‐ paddock systems, fast/slow rotation represent two to four or four to eight days per grazing event, while high/low stocking rates were 13.6 or 8.8 DSE ha⁻^1^, respectively. Bars represent grazing treatments, circles represent antimethanogenic feed additives. Other grazing and feed management are not shown.

**Figure 6:**
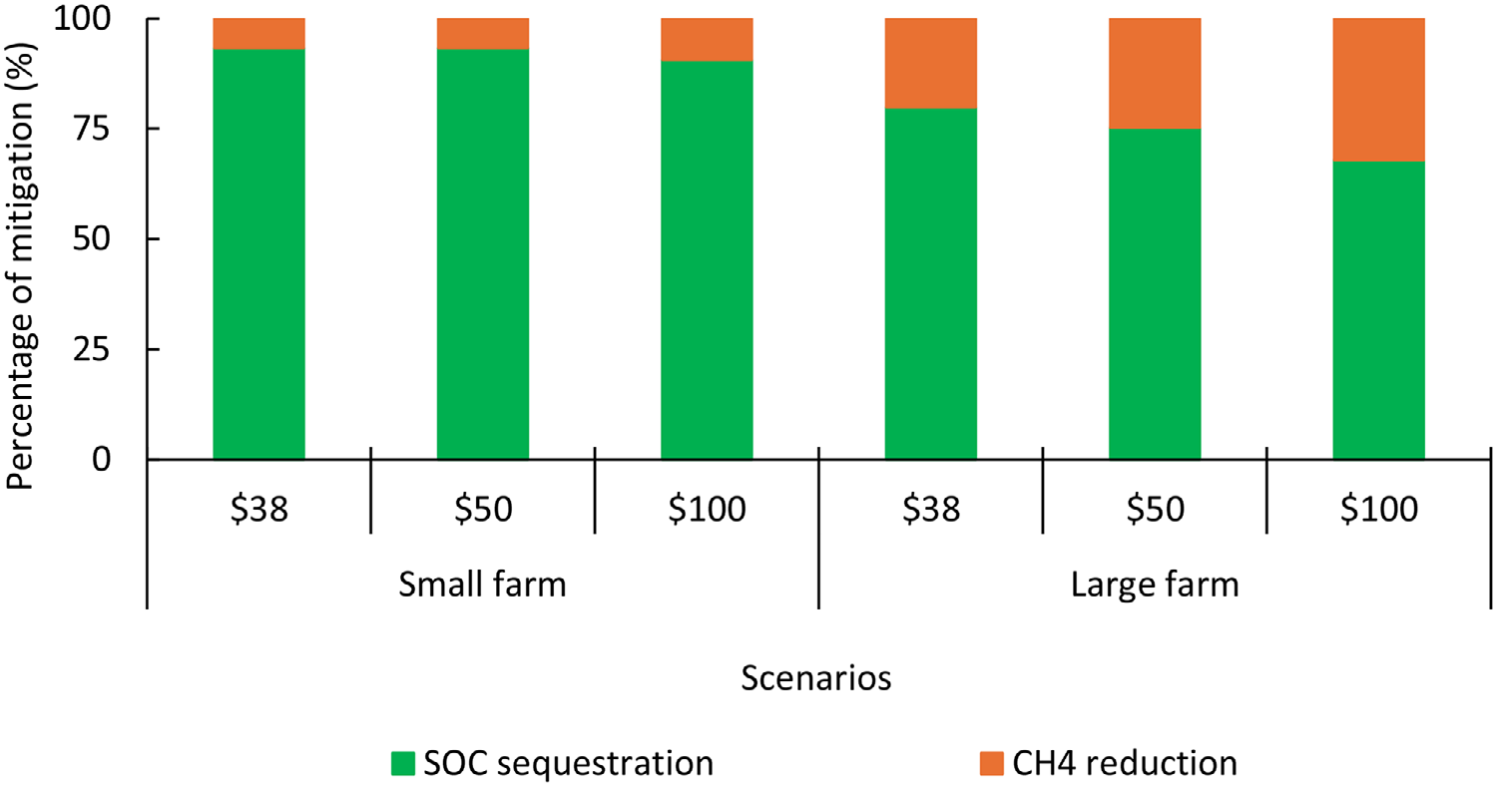
Trade‐offs between mean percentage GHG mitigation from SOC accrual and enteric methane reduction over ten years with variation in carbon price. Results drawn from optimal grazing and antimethanogenic feed additive management of Model I when all inputs remained constant, and two carbon price scenarios ($50 and $100 per t CO_2_‐e) were compared with initial carbon price ($38 per t CO_2_‐e).

Optimal grazing management regimes were similar across farm scales. Since SOC sequestration accounted for a significant proportion of GHG mitigation (**Figure** **6**), annual optimal grazing management was primarily dictated by SOC flux in prior years. Optimal results for Model I and II often reflected 15 paddock systems with high stocking rate and fast rotation, with splitting of grazing area to treatments of fast or slow rotation in final years (**Figure** **5**). High stocking rate and fast rotation increased flock size and sheep revenue, but SOC accrual decreased from years six to year eight. Thus, the combination of 15‐ and 30‐paddock treatments were optimal in years nine and ten to improve SOC.

When the objective was to minimize net GHG (Model III) on the small farm, optimal grazing management shifted from 15 paddocks with slow rotation and low stocking rate to flexible grazing in year 4, before reverting to the original system in year 5, then splitting farm area between flexible grazing with treatments of low and high stocking rates from year 9 onward (Figure 5). Although SOC accrual increased, optimal grazing treatments were different in year 4 due to a decrease in enteric methane reduction and an increase in GHG emissions in the preceding years. In year five, flock size decreased when cast‐for‐age ewes were sold, resulting in GHG mitigation and a reversion of optimal grazing treatments. Multiple grazing treatments were selected in years 9 and 10 to respond to a decrease in SOC stocks from year 6 to year 8. Optimal antimethanogenic feed additives (Figure 5) and time of sheep sales in Model I also differed from those of Model II and III. Sheep were sold at 21 months in Model I, 22 months in Model II, and 18 months old in Models III.

There were similar trends in profit from sheep sales, environmental income and emissions intensity on average and annually between two farm scales although average profit of Model I for the large farm was $10 DSE⁻^1^ greater and emissions intensity was 2.6 t CO_2_‐e DSE⁻^1^ higher than those of the small farm. Thus, for Model I, emissions intensity for the small farm was closer to the minimum net GHG (Model III) compared with those of large farm.

Similar trends in the number of grazing treatments were exhibited for Model I for the large farm, but a greater proportion of farm area was allocated to the 30 paddock system with fast rotation and high stocking rate in year ten (Figure 5). For Model III, the 15 paddock–slow rotation and low stocking rate or flexible grazing dominated grazing area of the large farm over 10 years, while a smaller area was allocated to the 15 paddock system with high stocking rate. Compared with the small farm, sheep production on the large farm emitted more GHG (Figure 3), underscoring need for greater mitigation. For Model III, management on the large farm responded more rapidly to increased GHG emissions compared with the small farm. Similar to results for the small farm, there was a split from year nine (due to a reduction in SOC from year 6 to 8) despite a larger area allocated to low stocking rate treatments and a smaller area to high stocking rate treatments (Figure 5). Implementation of antimethanogenic feed additives for all optimization approaches for the large farm was similar to those of small farm, except sheep were sold at the same age for Models I and II.

### 3.4 Drivers of Optimal Grazing Management and Antimethanogenic Feed Additives

#### 3.4.1 Sheep Price and Feed Costs

**Figure 7:**
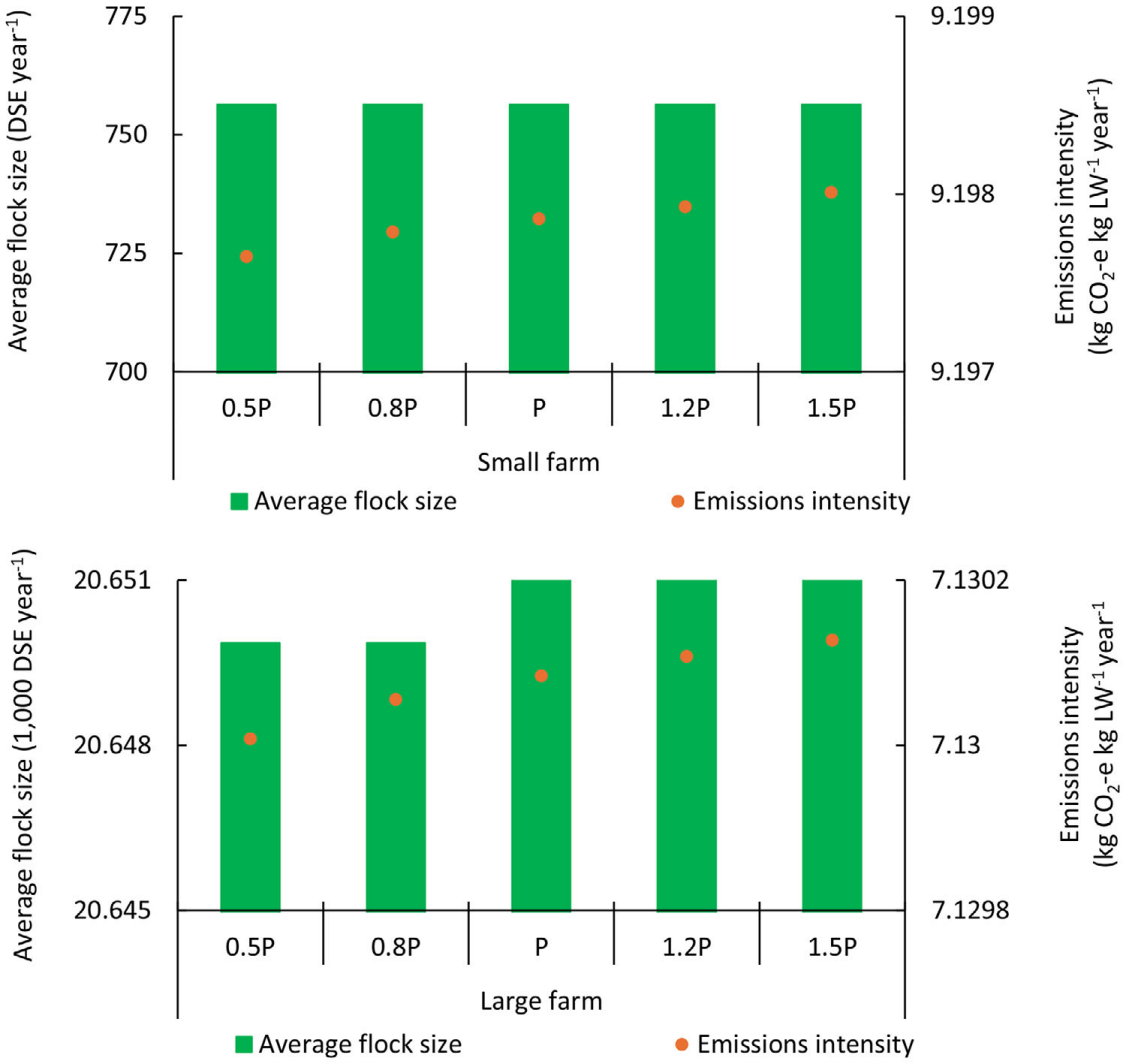
Impacts of sheep price on mean emissions intensity and average flock size. P represents baseline sheep price, while 0.5P, 0.8P, 1.2P and 1.5P represent four scenarios with reduced/increased price. Bars (flock size) relate to the left axis and circles (emissions intensity) relate to the right axis.

Although sheep price did not affect sale time or optimal antimethanogenic feed additives, sheep price influenced optimal grazing treatments and emissions intensity. The small farm tended to maintain flock size regardless of sheep price whereas lower sheep price reduced flock sizes for the large farm (**Figure** **7**). Emissions intensity for the small farm decreased slightly when sheep price went down and vice‐versa (Figure 7). Although a similar trend was observed for the large farm (Figure 7) when sheep price increased, emissions intensity increased by 0.19 kg CO_2_‐e DSE⁻^1^ when sheep price was reduced by 20%. Perturbation in emissions intensity for the large farm was greater than that of the small farm across most price scenarios, except for when prices were reduced by 50%. However, variability in grazing adoption was smaller when sheep price increased by 50% (compared with a 20% increase). Similar trends were evident for large farm, with adoption decisions changing slightly from year 7 onward when sheep price increased by 20%, but little difference when sheep prices were increased by 50%. When sheep price decreased, variability in the proportion of grazing area from year 1 was low, but significant differences emerged by year 10, as the farm reduced grazing area allocated to the 30 paddock system and increased area allocated to the 15 paddock system.

To examine implications associated with feed cost on optimal management decisions, we quantified how a 25–75% perturbation in pasture labor and supplementary feed costs impacted results. When supplementary feed costs were reduced by 25–75%, results changed only marginally, with shifts from 15 to 30 paddock systems on both farms in years nine and ten, and increased emissions intensity by 0.03–0.14 kg CO_2_‐e DSE⁻^1^ on the small farm and 0.04–0.16 kg CO_2_‐e DSE⁻^1^ on the large farm, respectively. When supplement cost was increased by 25% or 75%, the proportion of grazing area subjected to 15 paddock systems with high stocking rate increased slightly from year 8 to year 10, while emissions intensities decreased by 0.03–0.07 kg CO_2_‐e DSE⁻^1^ on the small farm or by 0.01–0.06 kg CO_2_‐e DSE⁻^1^ on the large farm. Similar trends were observed when labor costs associated with pasture were changed. A 25% or 75% reduction in pasture cost increased emissions intensity by 0.22–0.95 kg CO_2_‐e DSE⁻^1^ on the small farm, and by 0.15–0.74 kg CO_2_‐e DSE⁻^1^ on the large farm, respectively. An increase in pasture labor costs by 25% or 75% reduced emissions intensity by 0.15–0.37 kg CO_2_‐e DSE⁻^1^ on the small farm and by 0.11–0.16 kg CO_2_‐e DSE⁻^1^ on the large farm, respectively. Overall, these results indicate that assumptions associated with supplementary feed and pasture costs on GHG emissions were relatively small, suggesting that our interpretations are robust.

#### 3.4.2 Carbon Price

**Figure 8:**
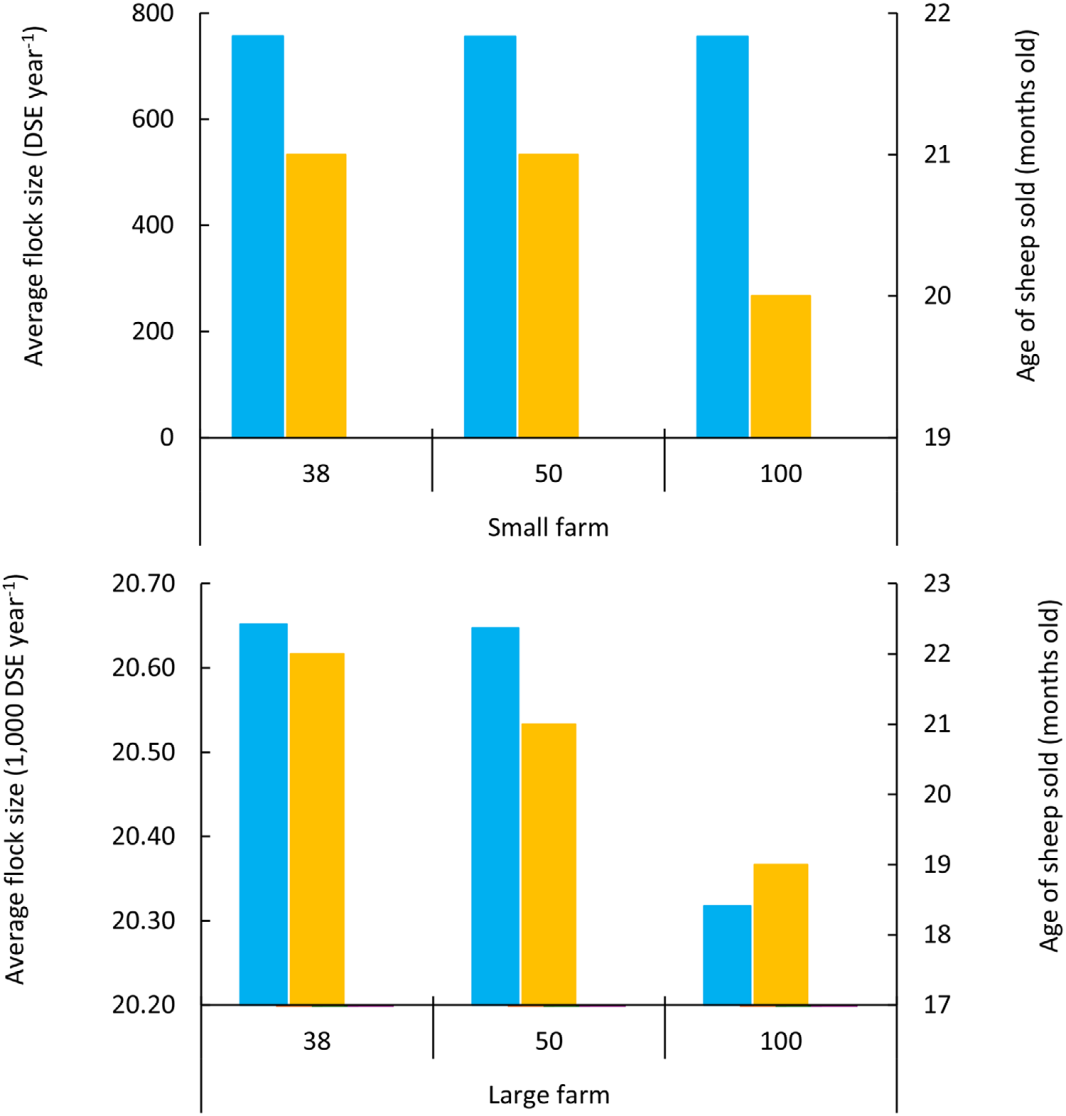
Impact of carbon price on average number and age of sheep sold. Results are plotted for Model I for carbon prices of $38 per t CO_2_‐e and two future price scenarios ($50 and $100 per t CO_2_‐e). Blue bars represent average flock size, yellow bars represent average sheep age.

Carbon price was a key driver of optimal grazing management to mitigate GHG emissions. As carbon prices increased, the proportion of grazing area subject to high stocking rate treatments decreased, while those subject to flexible grazing or low stocking rate treatments increased, with more prominent changes for the large farm when carbon price was $100 per t CO_2_‐e. Optimal management for the small farm shifted from the 15 paddock system to the 30 paddock system when carbon price was $100 per t CO_2_‐e. Flock size tended to remain constant on the small farm regardless of carbon price, while the optimal number of sheep on the large farm decreased when carbon price was $50 and $100 per t CO_2_‐e (**Figure** **8**), leading to reductions of ≈0.009 and 0.047 t CO_2_‐e DSE⁻^1^ in emissions intensity, respectively.

## 4 Discussion

The aim of this study was to identify optimal combinations of grazing management and antimethanogenic feed additives that maximized profit and/or minimized GHG emissions, and examine how these relationships varied across farm scales. The 15 paddock and 30 paddock systems with high stocking rate most often simultaneously maximized profit and minimized GHG emissions, regardless of farm scales. The 0.5% *Asparagopsis* supplementation often resulted in the lowest net GHG emissions, but the cost of feeding *Asparagopsis* was higher than that associated with feeding 3‐NOP or biochar. Although 3‐NOP evoked lower GHG mitigation, 3‐NOP resulted in greater profit due to lower cost. Taken together, the 15 and 30 paddock high stocking rate system with *Asparagopsis* was often the optimal management for balancing economic and environmental goals (Model III).

We showed that high stocking rates for a long period reduced pasture productivity, decreased below‐ ground biomass and organic matter inputs to soils, leading to SOC loss and erosion, similar to results hitherto.^[^ ^77^, ^80^, ^81^ ^]^ In contrast, lower stocking rates^[^ ^82^, ^83^ ^]^ in combination with rotational grazing^[^ ^84^, ^85^ ^]^ reduced pressure on pasture, increased regrowth vigor and promoted greater SOC accrual.^[^ ^11^, ^86^, ^87^ ^]^ In line with our results, other studies have shown that multi‐paddock grazing systems^[^ ^31^, ^88^ ^]^ and high density short term rotational grazing^[^ ^89^, ^90^, ^91^, ^92^ ^]^ can optimize pasture utilization and improve SOC and total nitrogen compared with continuous grazing.^[^ ^93^, ^94^ ^]^ Our results underscore the importance of sequestration duration with multiple interventions for long‐term and continued carbon removals. While effects of weather were consistent for all treatments, recent studies suggest that extreme weather events can reduce pasture production, increase soil respiration, and increase SOC loss.^[^ ^70^, ^95^, ^96^, ^97^ ^]^

We found that antimethanogenic feed additives were less adoptable compared with changes to grazing management (Figure 6). Antimethanogenic feed additives, such as *Asparagopsis* and 3‐NOP, realized deep cuts in enteric methane^[^ ^60^, ^61^, ^62^ ^]^ although high costs^[^ ^98^ ^]^ and logistic difficulties in sourcing and administering additives can inhibit adoption of such feed supplements in extensive‐grazing systems. Biochar, in contrast, offers lower methane reduction potential,^[^ ^49^, ^58^, ^59^ ^]^ but is more affordable and potentially more functional, having modest antimethanogenic properties while serving as a soil organic amendment. These results highlight trade‐offs between per unit DM intake, mitigation and cost, with e.g., *Asparagopsis* having higher methane mitigation potential but costing more, and with biochar having lower potential for methane inhibition, but lower cost. Construction of marginal abatement cost curves that contrast trade‐offs between cost and GHG mitigation for optimized management regimes would seem fruitful in future research. The potential of machine learning with process‐based models suggests that more combinations of management can be compared than ever before.^[^ ^99^ ^]^ Such analyses could account for wider benefits of mitigation interventions, such as the impacts of biochar enrichment of manure on soil fertility and ensuing pasture production.^[^ ^96^ ^]^

Enteric methane mitigation efficacy associated with antimethanogenic feed additives could be further influenced by sheep liveweight, as heavier animals tended to produce more enteric methane^[^ ^100^ ^]^ due to greater feed intake. As such, breeding animals (the core ewe flock in the present study) often produce a greater proportion of methane than do young animals. At higher dose rates, we found that such antimethanogenic feed additives significantly reduced farm methane emissions. As well, selling sheep at an earlier age can avoid methane, although this tends to have less relative impact on net farm GHG compared with feed supplements because breeding animals produce larger proportions of farm methane.^[^ ^5^, ^77^ ^]^ Adoption of antimethanogenic feed additives, in combination with contextualized management of production efficiency, sheep age and feed sources, afford enteric methane mitigation while enhancing farm sustainability. Another mechanism worthy of further investigation would be anti‐methanogenic pasture species, such as leucaena, Desmanthus, or birdsfoot trefoil,^[^ ^6^, ^101^ ^]^ as such species may come with wider benefits, assuming pastures were able to proliferate in the desired environment and were conducive to animal production.^[^ ^102^ ^]^

Consistent with previous studies,^[^ ^4^, ^61^, ^103^, ^104^ ^]^ we found that stacking mitigation options could realize greater benefits than any single management intervention, with lower emissions intensity realized when interventions were bundled. However, SOC sequestration accounted for larger proportion of mitigation strategy compared with enteric methane reduction (Figure 6). Thus, multiple grazing treatments combined with one antimethanogenic feed additives were optimal for small and large farms. We note significant variation in longitudinal emissions intensity, e.g., attaining its lowest point in year six (Figure 4); because ewes were cast for age in the preceding year (Figure 1), resulting in significant meat production to offset farm level GHG.

Optimal combinations of grazing and feed management vary depending on farm management strategy and farm scale. When profit was key goal, farms tended to adopt 15 paddock system with fast rotation and high stocking rate without using feed additives to enhance animal productivity, reduce feed costs, and increase profit. Although fast rotation and high stocking rates dominated grazing area in most years due to greater profit, there was an alternation of 15 and 30 paddocks with fast and slow rotations in years 9 and 10 to improve SOC sequestration. When reducing net GHG emissions was the primary motivation, flexible grazing or 15 paddocks with slow rotation and low stocking rate were optimal on large farms. In contrast, optimal management on small farms was often high and low stocking rate treatments, primarily relying on 15 paddocks with high stocking rate. Emissions from large farms were higher than those from small farms due to larger flock size, indicating need for greater mitigation. There were also shifts between 15 paddocks with slow rotation low stocking rate and flexible grazing in a few years, reflecting changes in SOC and enteric methane reduction, and the dynamic nature of balancing flock size with an emission mitigation goal. Flock size was adjusted during that period to accommodate ewe replacement, ensure optimal land use, and minimize net emissions overall. While some of our optimal solutions altered between 15 and 30 paddock systems from one year to the next, these may be less practicable because farmers would not change permanent fencing infrastructure from one year to the next unless they used temporary electric fences that were amenable to agile change.

Treatments with high stocking rate often dominated regardless of farm size because stocking rates had greater influence on profit than revenue from carbon, similar to results seen elsewhere.^[^ ^105^, ^106^, ^107^, ^108^ ^]^ As fast rotation and high stocking rate were detrimental to SOC in the long term,^[^ ^70^ ^]^ a combination of the 15‐ and 30‐paddock systems with slow rotation tended to be adopted in years 9 and 10 to reduce pressure on pasture, improve regrowth and soil health. Compared with the 15‐paddock system, the extended rest duration for the 30‐paddock system fostered deeper root growth and more vigorous pasture regrowth, enhancing biomass production, reducing risk of soil compaction, and improving SOC accrual.^[^ ^19^, ^23^ ^]^ Despite greater impacts in minimizing net emissions, inclusion of 0.25% dietary *Asparagopsis* was adopted less frequently due to higher cost. Instead, inclusion of 0.2% dietary 3‐NOP was often more favorable due to lower cost per unit emission reduction.

Sheep price was a key driver of optimal grazing and feed management. When sheep price decreased, small farms reduced feed cost and focused on emissions abatement for environmental income. Despite no differences in flock size, grazing management or selling time when sheep price decreased, their emissions intensity was reduced due to reduced pasture biomass and increased consumption of antimethanogenic feed supplements. In contrast, optimal results for large farms often comprised shifting from the 30‐ to the 15‐paddock system to reduce production costs while adjusting flock size to mitigate net emissions and increase environmental income. When sheep price increased, there were more changes in grazing management on the small farm compared with the large farm. Higher sheep prices may encourage smaller farms to take on more risk by intensifying their production, aiming to maximize potential rewards relative to their baseline income. In contrast, larger farms may prioritize stability and long‐term sustainability over making significant changes in response to short‐term market trends.

Feed costs, such as costs of supplementary feeds or labor costs associated with pasture management, only had marginal effect on optimal grazing regimes compared with the larger effects of flock size, sale time, and feed management. This indicates that even with larger differences in feed costs, our interpretations with respect to optimal farm management decisions would be similar, suggesting that our conclusions are robust. When alternative feed costs were used, optimal decisions first impacted grazing management and paddock configuration, probably because grazing regime influenced total feed use (such as pasture utilization and supplement reliance) and land productivity. When feed costs decreased, optimal decisions favored profit by intensifying production to capture increased revenue. Conversely, higher feed cost engendered optimal grazing regimes more focused on environmental stewardship, assuming farmers were supported by incentive schemes that reward lower emissions intensity. Decisions on flock size, sale time, and optimal feed management were more responsive to market prices, seasonality and target weights rather than feed costs.

Consistent with carbon price scenarios articulated by our previous study,^[^ ^70^ ^]^ we found that farms adjusted their grazing management strategies, selling schedules and flock size to reduce net emissions and enhance environmental income depending on market conditions. Carbon prices had greater influence on GHG emissions reduction than sheep price or supplementary feed and pasture costs. Compared with sheep prices, carbon prices evoked greater change in grazing management on large farms due to greater land area, financial capacity, and operational flexibility. Smaller farms face greater constraints to land area and economic capital, which may restrict their ability to participate in carbon markets and realize financial benefits. Greater government incentives to support small farm business participation in carbon markets may encourage wider adoption of multiple concurrent mitigation options, helping level the playing field between small and large producers.

Across farming systems, mitigation options and carbon prices examined, profit from sheep production significantly outweighed income from GHG emissions reduction. Consistent with previous work,^[^ ^109^ ^]^ our results indicate that sheep production systems premised on pasture grazing are unlikely to become net carbon sinks under current commodity and carbon pricing. E.g., when the carbon price used in Model I was $100 per t CO_2_‐e, sheep profit was ≈5 or 12 times greater than environmental income derived by the small and large farm, respectively. Despite modest economic incentives to alter management decisions toward GHG abatement, our results demonstrate that the ratio of carbon price to livestock prices (for meat and wool) to drive transformational uptake of carbon farming. Hence, climate policies may need to be coupled with other instruments to engender change, such as subsidies, mandates or payments for ecosystems services to improve the value proposition of carbon farming.

## 5 Conclusions

A foundational insight of our study is that farmers are likely to alter management practices if the cumulative economic and environmental benefit outweighs the cumulative economic and environmental disadvantage associated with adoption, noting that most changes to farming systems evoke co‐benefits and trade‐offs. We posit this conclusion would extend to social, psychological, and institutional dimensions. We found that profit was highest for multiple paddock systems with high stocking rates and short grazing periods whereas net GHG emissions were lowest for multi‐paddock low stocking rate systems with 0.5% *Asparagopsis* feed supplementation. This difference reflects tensions between profitability and GHG emissions reduction, as stocking rates improve profit but also tend to increase GHG emissions per area, while low stocking rate and antimethanogenic feed additives reduce emissions but lower profit. However, management practices affording low GHG emissions and higher profit than baseline systems were achievable: high stocking rate fast rotation systems using 3‐NOP feed additive could balance high productivity with moderate GHG emissions reduction. Grazing management for SOC sequestration had a greater impact on emissions abatement than use of antimethanogenic feed additives due to greater income from SOC, productivity co‐benefits associated with pasture growth and more persistent benefits from practices that improved SOC. In contrast, antimethanogenic additives had to be fed on a daily basis to emanate continued mitigation and thus attracted daily costs. As carbon prices increased however, relative carbon farming income from enteric methane mitigation increased, particularly for larger farms.

We found that higher carbon prices may catalyze participation in carbon markets and help mitigate farm enterprise GHG, but relative benefit depends on farm size, existing land condition, and ability of animals to regularly consume antimethanogenic feed supplements. Large farms were better positioned to implement carbon farming measures, with access to more land, financial resources, and operational flexibility. Income from carbon farming can diversify revenue streams, potentially improving profitability under drought, as most income derived from livestock enterprises in Australia hinge partially or completely on receiving consistent seasonal rainfall. On the other hand, small farms may face greater challenges to participating in carbon markets due to higher transaction costs and administrative barriers associated with certification. If carbon markets became more accessible and inclusive through government subsidization, aggregation of projects across farms, or higher payments for co‐benefits associated ecosystem services, small farms may be more amenable to participation. As such, while the carbon market has potential to drive long‐term societal benefit, the significantly higher ratio of commodity to carbon prices currently incentivizes producers to prioritize livestock production over carbon farming. Since we grounded assumptions on data from two real sheep farms, our optimal management regimes may not apply to all production systems and agroecological regions (as this was not the aim of the study). Future research could thus examine how optimal management with a profit or mitigation lens varies across farm sizes, environments, management practices, and enterprise types. Construction of marginal abatement cost curves that contrast trade‐offs between cost and GHG mitigation for optimized management regimes would also seem fruitful, as would elucidation of how the relative balance between income from carbon farming and livestock farming vary across agroecological regions. This would deepen insight into those areas better placed for supporting food security, and those better prioritized for carbon farming and provision of ecosystems services, such as conservation of biodiversity habitat.

## Author Contributions

M.P.‐K. and M.T.H. wrote the first draft; M.P.‐K. conducted farm systems simulations and linear programing; M.T.H. and M.P.‐K. conceptualized the study; all authors refined the study design and revised the manuscript.

## Conflict of Interest

The authors declare no conflict of interest.

## Supporting information



Supporting Information

## Data Availability

The data that support the findings of this study are available from the corresponding author upon reasonable request.
