# Supplementary material for: Stacking Interventions Enhances Carbon Removals and Profitability of Livestock Production Systems
Source: Adv Sci (Weinh). 2025 Jun 25;12(33):e03382. doi: 10.1002/advs.202503382 (PMC12412474; doi:10.1002/advs.202503382)
Supplement: Supplementary file 1 — Supporting Information [file ADVS-12-e03382-s001.docx]

**APPENDIX A. SHEEP PRICES AND PRODUCTION COSTS**

- Costs of sheep production included:

| Land management costs | - Repairs and maintenance, excluding fencing - Sprays and weed control - Pest control |
| --- | --- |
| Pasture management costs | - Sowing - Pasture renovation - Fertiliser - Irrigation, including water and electricity - Fuel, such as petrol, diesel, and oil |
| Costs of supplementary feeds | Including purchased feeds like hay, grain. |
| Stock management costs | - Animal health, such as drenching, vaccinations, medicines, veterinary services - Handling/contractors - Transport, such as freight and cartage - Marking - Shearing - Crutching - Breeding, including artificial insemination, scanning |
| Selling costs | - Brokerage =/commission - Levies |
| Fixed costs | - Insurance - Accounting and financing - Other overhead costs |
| Fencing cost | Fencing costs were calculated following economics analysis for different multi-paddock systems ^[3]^ |

- Sheep price: Baseline sheep prices were sourced from the small and large farm, which were $2.24 and $2.56 per kg liveweight, respectively.
